# Transcriptome datasets and histological profiles of critical larval stages in gilthead seabream

**DOI:** 10.1016/j.dib.2024.110571

**Published:** 2024-06-13

**Authors:** Babak Najafpour, Adelino VM Canário, Deborah M. Power

**Affiliations:** a Centro de Ciências do Mar (CCMAR), Universidade do Algarve, Faro, Portugal; b International Research Center for Marine Biosciences (Ministry of Science and Technology), Shanghai Ocean University, Shanghai, China

**Keywords:** Aquaculture, Early development, Flexion, Gilthead seabream, Mid-metamorphosis

## Abstract

The transcriptome of the seabream larvae farmed in different European commercial hatcheries was analysed during critical larval stages. The complementary data herein presented support the findings reported in the associated research article “Insights into core molecular changes associated with metamorphosis in gilthead seabream larvae across diverse hatcheries”. Samples were collected from gilthead seabream (*Sparus aurata*) hatcheries in Greece (site Gr), Italy (site It), and France (site Fr). RNA was extracted from larvae with different weights, mainly at the flexion (23 and 25 dph) and mid-metamorphosis stages (43, 50, 52, 56, and 60 dph). RNA-seq libraries were sequenced using Illumina HiSeq xten. The paired-end sequenced raw reads were deposited in the NCBI-SRA database with the accession number PRJNA956882. Differential expression and function of genes were obtained by comparing transcriptome profiles of larvae at different developmental stages. The presented data can be used to improve marine-farmed fish larvae production during critical larval stages.

Specifications Table

**Table:** 

Subject	Biological sciences
Specific subject area	Aquaculture; early development; flexion; gilthead sea bream; mid-metamorphosis
Type of data	Figures, Tables, Supplementary Tables Raw, Analyzed data, Filtered data, Processed data
Data collection	Gilthead seabream larvae at different developmental stages and of different quality was sampled from several aquaculture hatcheries and fixed in an appropriate fixative. RNA was extracted from gilthead seabream larvae using an E.Z.N.A. Total RNA Kit I. After quality control of RNAs using agarose gel electrophoresis and spectrophotometry, paired-ended RNA-seq libraries were constructed and sequenced on an Illumina HiSeq xten platform. Bioinformatics was performed on raw data for interpretation. In parallel, histological slides characterized larval development.
Data source location	Greece, Italy (Ionian Sea), France (Bay of Biscay)
Data accessibility	Repository name: NCBI, Zenodo Data identification number: PRJNA956882 DOI 10.5281/zenodo.10581938 Direct URL to data: https://dataview.ncbi.nlm.nih.gov/object/PRJNA956882?reviewer=t6u9iddqq15fbv9u3698ejko6b https://zenodo.org/records/10581939
Related research article	Insights into core molecular changes associated with metamorphosis in gilthead seabream larvae across diverse hatcheries. https://doi.org/10.1016/j.aquaculture.2024.740979

## 1 Value of the Data

• Mortality and low quality of larvae are the main bottlenecks of larval rearing in commercial aquaculture hatcheries. Transcriptome analysis of larvae batches with different qualities/weights/ages during critical larval stages (e.g., flexion and mid-metamorphosis) is valuable for assessing the biological capacity of the larvae at these stages and the factors affecting larval quality.
• Analysis of samples from different aquaculture sites identified common gene markers related to age/weight and reduced the bias derived from site-specific conditions.
• Both biologists and aquaculturists can benefit from the available datasets since the larval samples were collected from routine production cycles under hatchery conditions, and the transcriptome dynamics associated with age/weight was profiled.
• The transcriptome comparisons of the seabream larvae at different developmental stages provides insight into the biological capacity of this species. This information can help adjust management regimes to make them better suited to the physiology of seabream or other marine species larvae. Gene markers were identified that are involved in different biological processes (e.g., larvae nutrition or immune response).

## 2 Background

Mortality and low-quality larvae are the main bottlenecks of larval rearing in commercial aquaculture hatcheries. Transcriptome analysis of larvae batches with different qualities/weights/ages during critical larval stages (e.g., flexion and mid-metamorphosis) is valuable for assessing their biological capacity and may give insight into the biological basis of larval quality. Therefore, we designed a largescale study to collect larvae at different developmental stages across several gilthead seabream hatcheries in Europe (Greece, Italy, France) and analysed the transcriptome modifications using RNA-seq. We have highlighted the key observations in the related research paper (https://doi.org/10.1016/j.aquaculture.2024.740979). The present supplementary article is intended to serve as a data resource for biologists and aquaculturists seeking deeper insights into the biological capacity of larvae during critical developmental stages.

## 3 Data Description

**Fig. 1:**
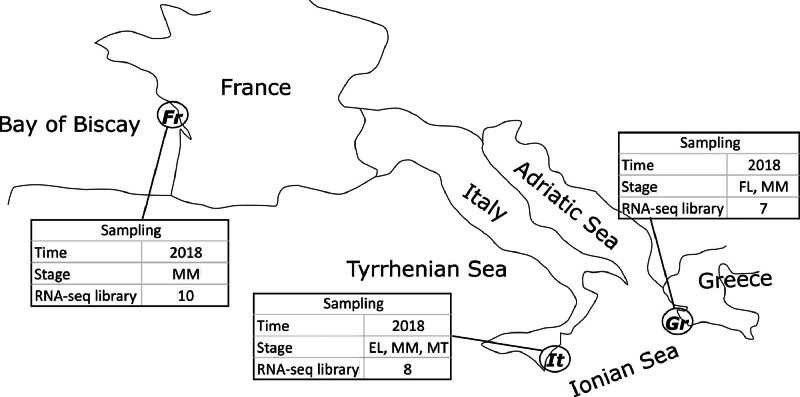
A scheme of the location and sampling of gilthead seabream from hatcheries. The time, larval stages (FL = flexion, El = end of larvae rearing, MM = mid-metamorphosis, MT = metamorphosis), and the number of RNA-seq libraries obtained from samples obtained from the three hatcheries (France, Fr; Italy, It; Greece Gr) are specified.

**Fig. 2:**
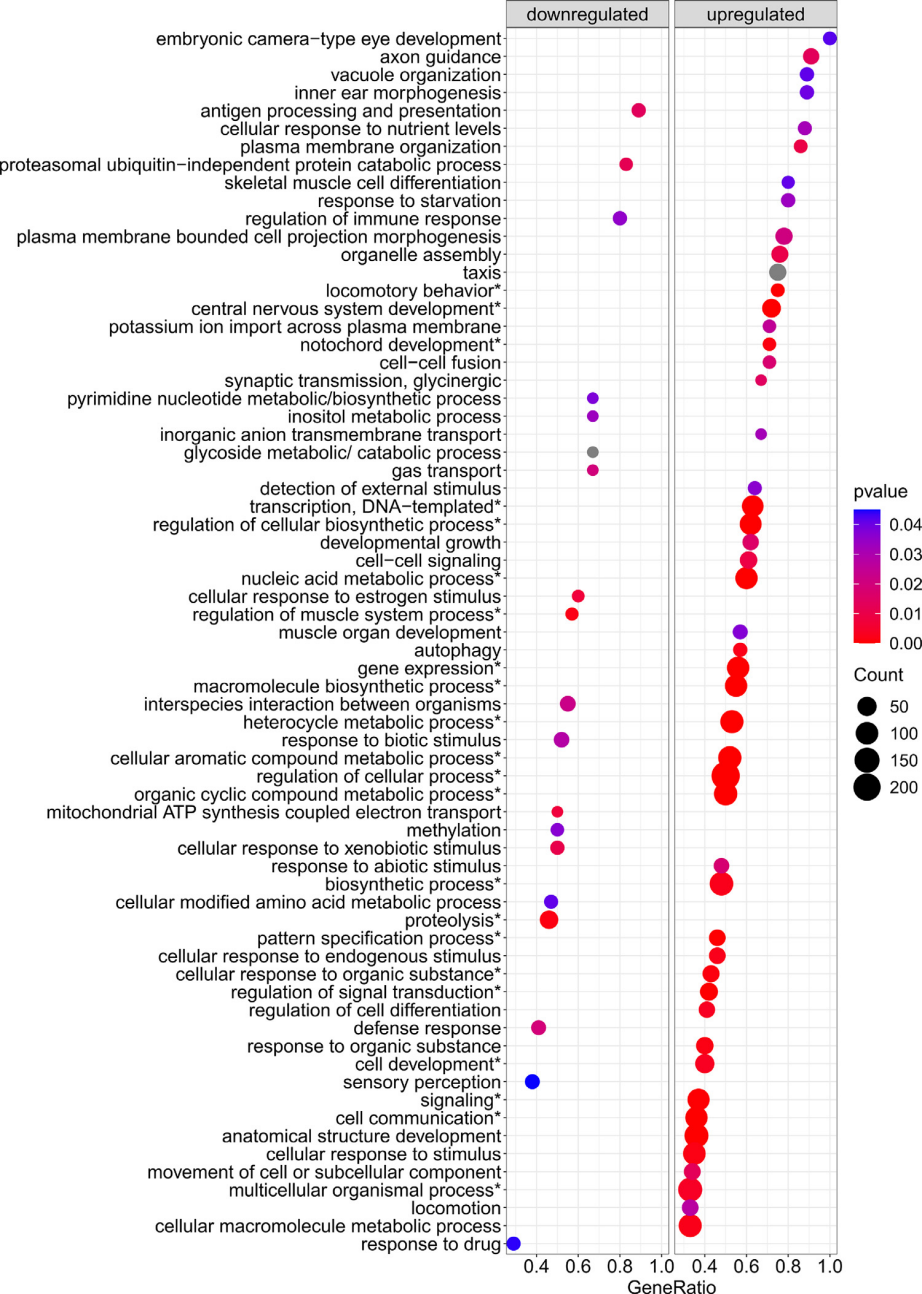
The enriched gene sets related to biological processes (*p-value* < 0.05) in gilthead seabream larvae at the flexion stage (24 dph) compared to mid-metamorphosis (51 dph) larvae. The gene set enrichment analysis was performed using the DEGs obtained when the transcriptome of the 24 dph and 51 dph larvae were compared.

**Fig. 3:**
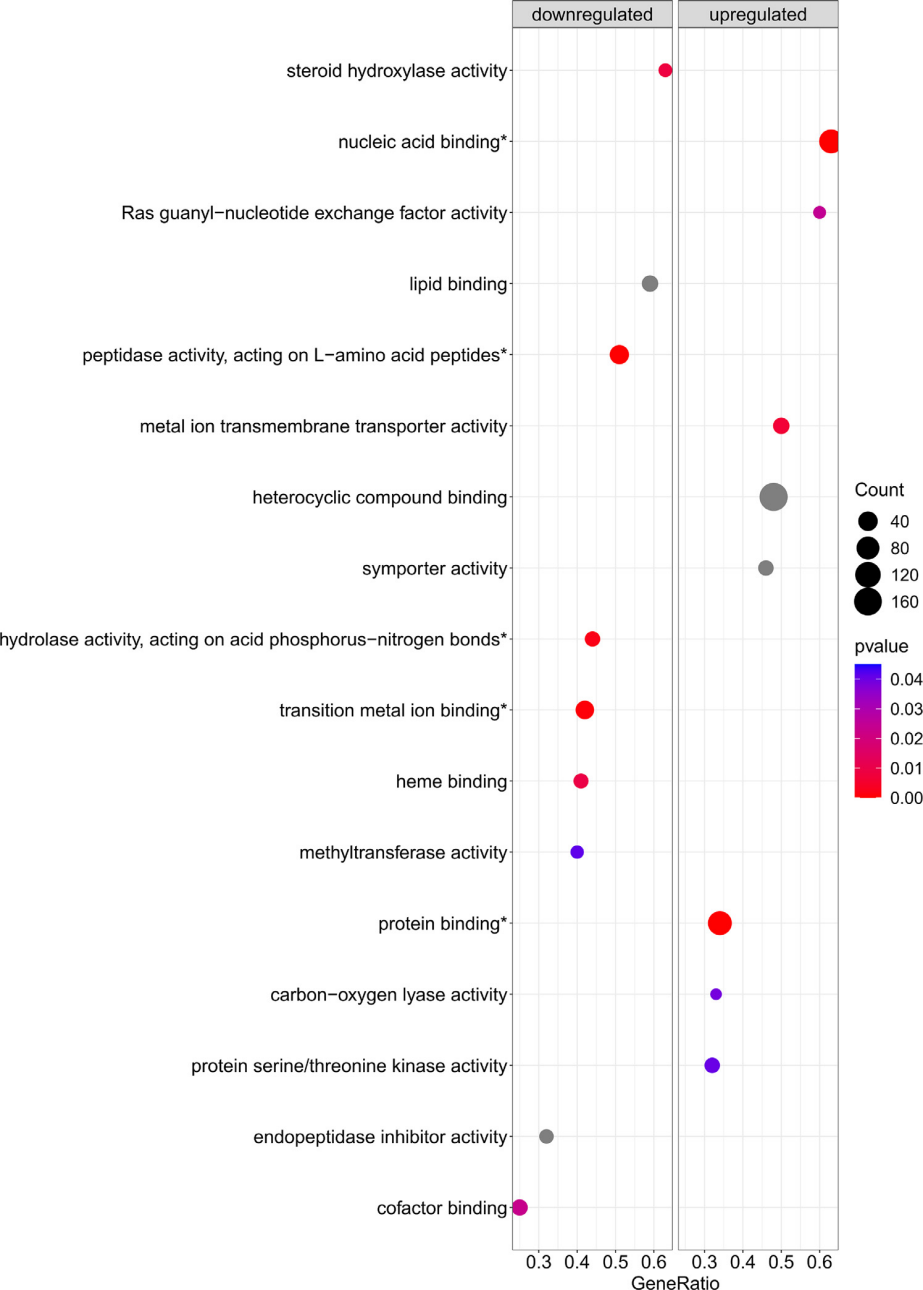
The enriched gene sets related to molecular function (*p-value* < 0.05) in gilthead seabream larvae at the flexion stage (24 dph) compared to mid-metamorphosis (51 dph) larvae. The gene set enrichment analysis was performed using the DEGs obtained when the transcriptome of the 24 dph and 51 dph larvae were compared.

**Fig. 4:**
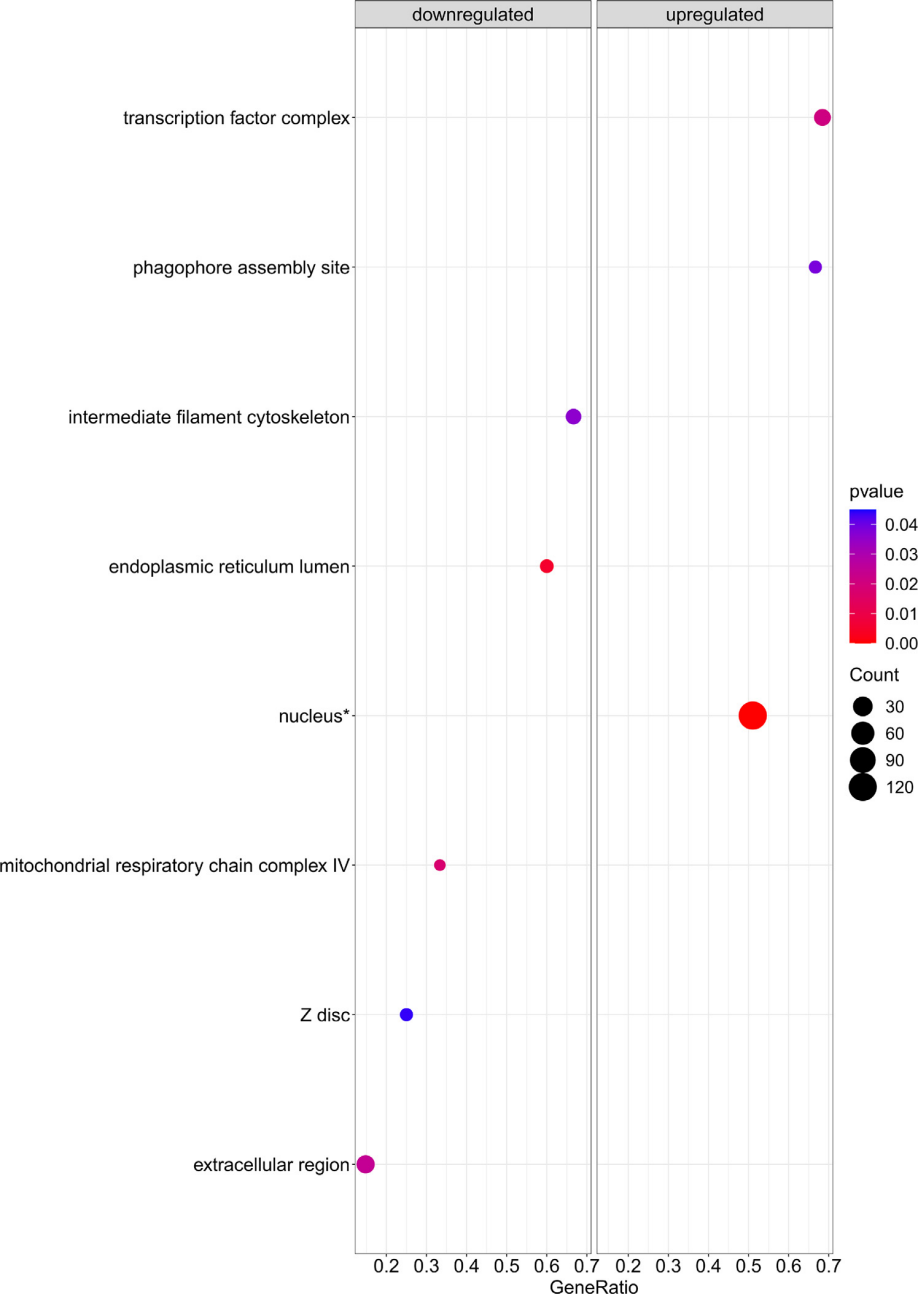
The enriched gene sets related to cellular components (*p-value* < 0.05) in gilthead seabream larvae at the flexion stage (24 dph) compared to mid-metamorphosis (51 dph) larvae. The gene set enrichment analysis was performed using the DEGs obtained when the transcriptome of the 24 dph and 51 dph larvae were compared.

**Fig. 5:**
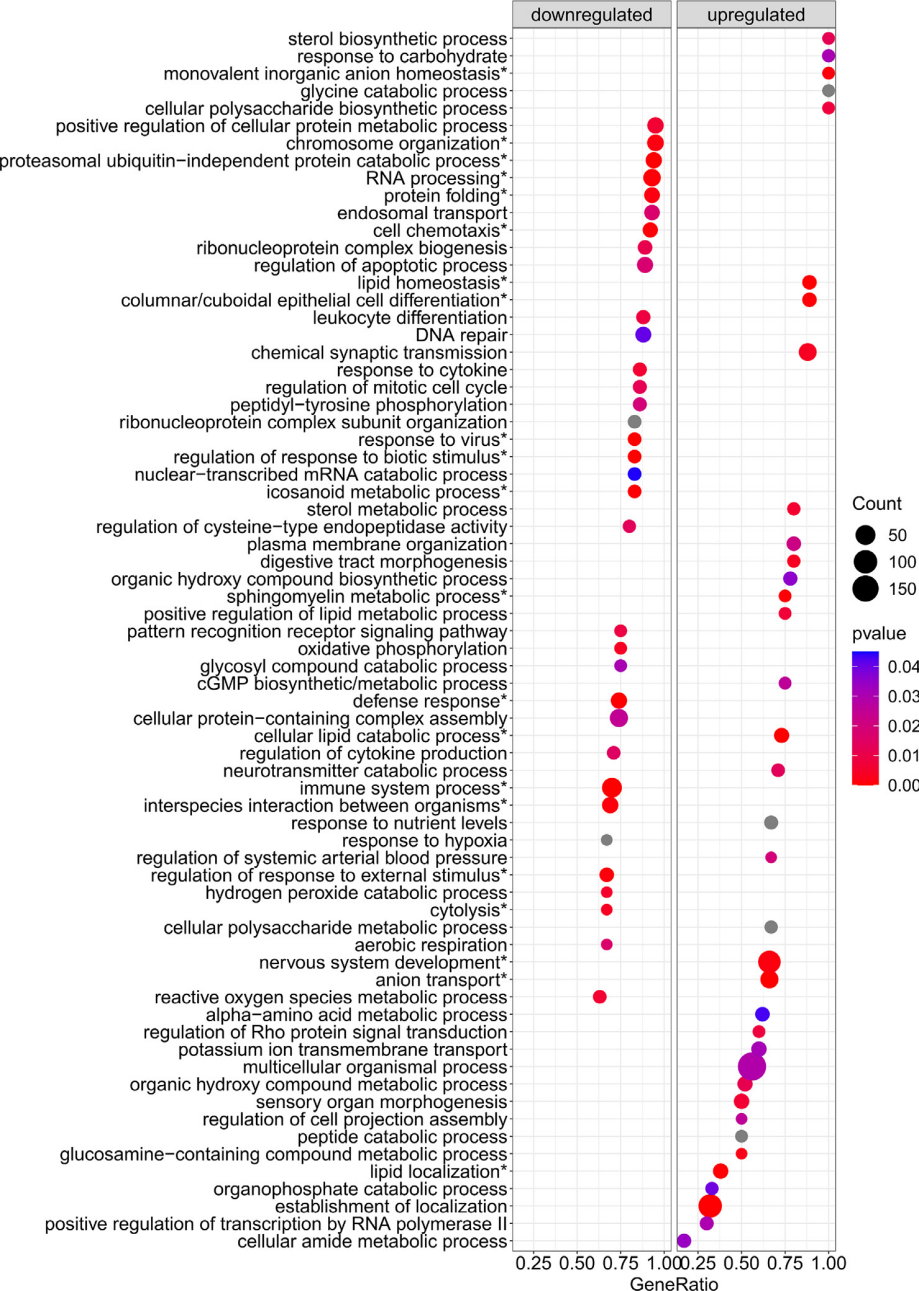
The enriched gene sets related to biological processes (*p-value* < 0.05) in gilthead seabream larvae at the mid-metamorphosis stage (46 dph). The gene set enrichment analysis was performed using the DEGs obtained when the transcriptome of the 46 dph and 54 dph larvae were compared.

**Fig. 6:**
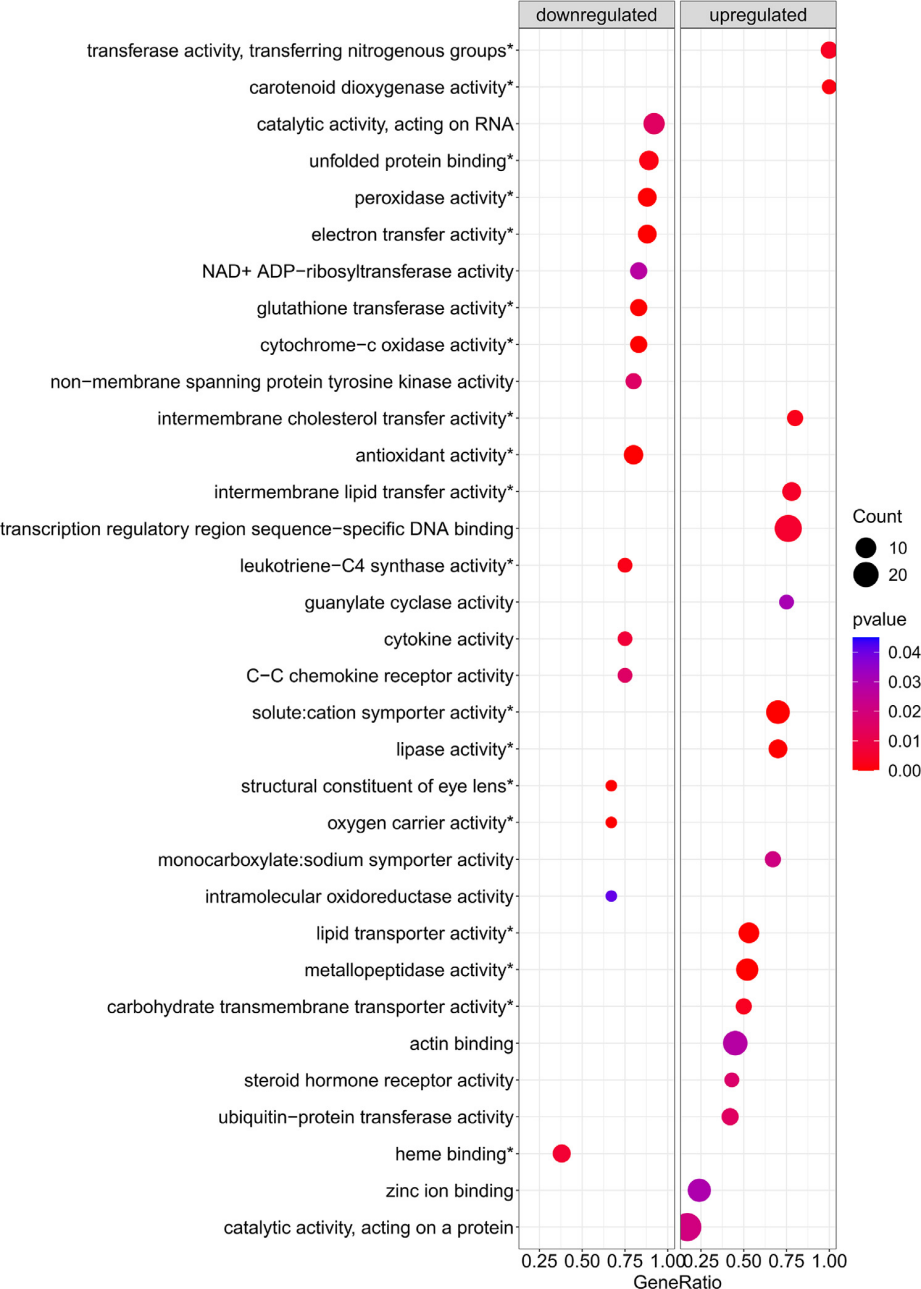
The enriched gene sets related to molecular functions (*p-value* < 0.05) in gilthead seabream larvae at the mid-metamorphosis stage (46 dph). The gene set enrichment analysis was performed using the DEGs obtained when the transcriptome of the 46 dph and 54 dph larvae were compared.

**Fig. 7:**
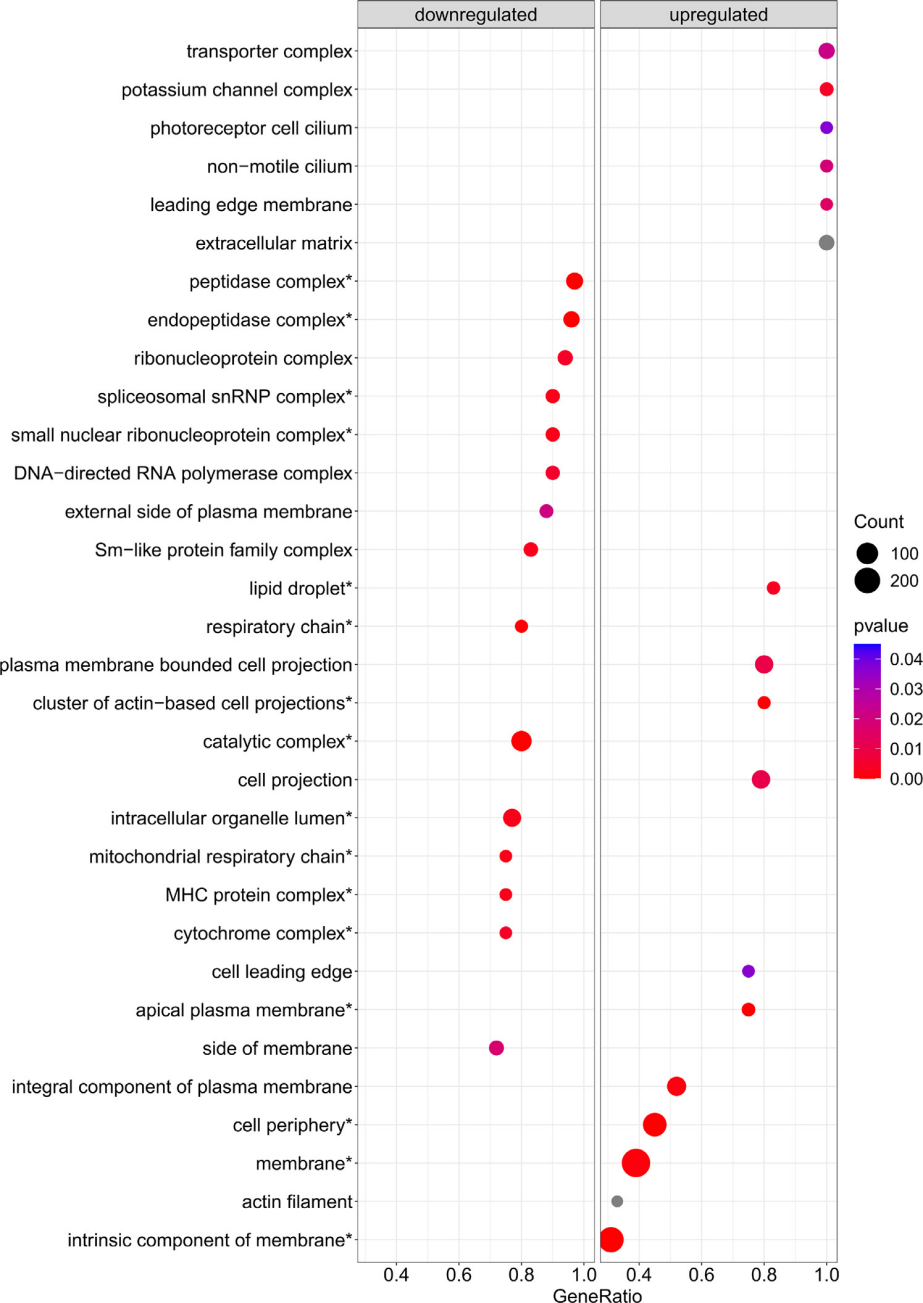
The enriched gene sets related to the cellular components (*p-value* < 0.05) in gilthead seabream larvae at the mid-metamorphosis stage (46 dph). The gene set enrichment analysis was performed using the DEGs obtained when the transcriptome of the 46 dph and 54 dph larvae were compared.

**Fig. 8:**
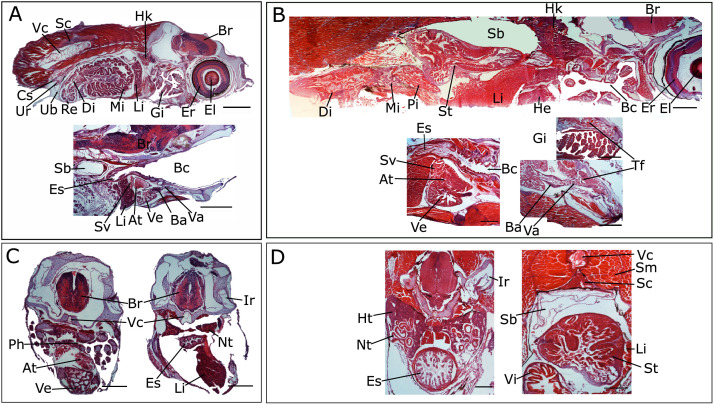
Sagittal (A, B) and transverse (C, D) histology sections of gilthead seabream larvae at flexion (A, C) and mid-metamorphosis (B, D). Larvae at flexion and mid-metamorphosis had an approximate age of 24 and 51 dph, respectively. The sections (10 µm thick) were stained using the haematoxylin and eosin (H&E) method [5]. Developmental changes in internal organs and tissues such as the heart, brain, stomach, intestine, kidney, swim bladder, gill, eye, vertebral column, liver, and skeletal muscle are obvious during the transition from flexion to mid-metamorphosis. Abbreviations: El, eye lens; Br, brain; Er, eye retina; Gi, gill; Li, liver; Hk, head kidney; Pi, proximal intestine; Mi, mid-intestine; Di, distal intestine; Re, rectum; Sc, spinal cord; Vc, vertebral column; Ub, urinary bladder; Ur, ureter, Cs, corpuscle of stannius; Bc, buccopharyngeal cavity; Sb, swim bladder; He, heart; St, stomach; Va, ventral aorta; Ba, bulbus arteriosus; Ve, ventricle; At, atrium; Sv, sinus venosus; Es, esophagus; Tf, thyroid follicles; Ir, inner ear; Ph, pharynx; Ht, hematopoietic kidney; Nt, nephron tubule; Sm, skeletal muscle; Vi, intestinal villi. Scale bar of sagittal sections (A, B) = 400 µm; Scale bar of transverse sections (C, D) = 200 µm.

**Fig. 9:**
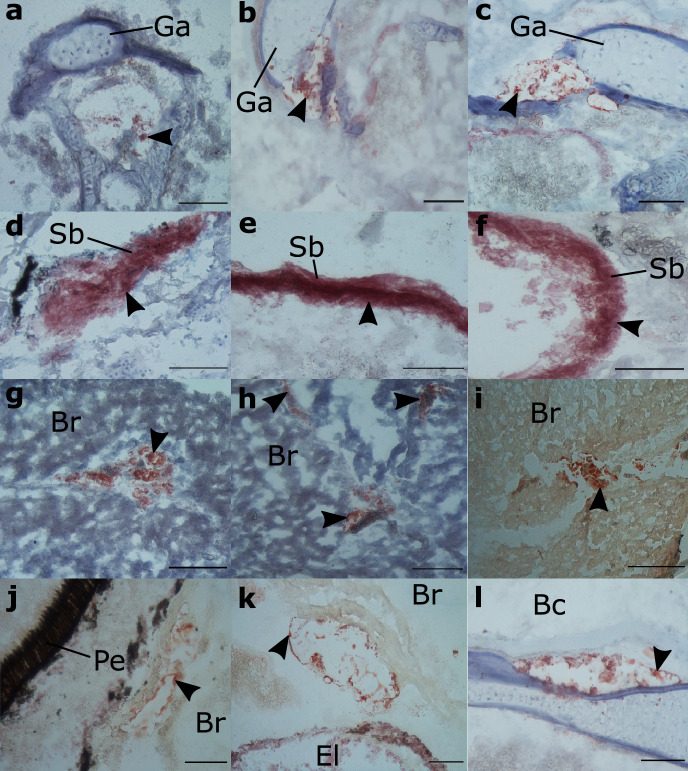
Lipid staining of sagittal sections of gilthead seabream larvae using Oil Red O on cryostat sections. Arrows indicate lipid droplets. Images **a – c** corresponds to gilthead seabream larvae at mid-metamorphosis (approximate age 50 dph). Image **d** identifies lipid droplets in gilthead seabream larvae at flexion (approximate age of 24 dph). Except for the swim bladder (Sb), which in larvae at flexion and mid-metamorphosis had clear lipid deposition, the deposition of lipids was most evident in mid-metamorphosis gilthead seabream larvae in the gill arch (Ga) and brain (Br).

Fig. 1 is a scheme showing the sampling locations and the number of RNA-seq libraries prepared from the larval samples collected from three gilthead seabream hatcheries in France (Fr), Italy (It), and Greece (Gr). Fig. 2, Fig. 3, Fig. 4, Fig. 5, Fig. 6, Fig. 7 present the enriched genes obtained from differential expression analysis of gilthead seabream larvae at different developmental stages (*p*-value < 0.05). Fig. 8 shows the histology of gilthead seabream larvae at flexion and mid-metamorphosis using histological sections and H&E staining. Fig. 9 characterizes the lipid deposition in gilthead seabream larvae at flexion and mid-metamorphosis using Oil Red O staining of cryostat sections.

Table 1 provides the average age and weight of the gilthead seabream larvae collected from several aquaculture hatcheries and used for RNA-seq analysis. Table 2 represents sequence statistics for 25 paired-end RNA-seq data mapped to the gilthead seabream reference genome. Table 3 provides the top 40 up- and down-regulated gene transcripts in gilthead sea bream larvae at the flexion stage (24 dph) compared to 51 dph larvae (*p-value* < 0.05). Table 4 provides the top 40 up- and down-regulated gene transcripts in gilthead seabream larvae at the mid-metamorphosis stage (46 dph) compared to 54 dph larvae (*p-value* < 0.05).

**Table 1:** The average age and weight of gilthead seabream larvae used for RNA-seq analysis across several aquaculture sites.

RNA-Seq library	Number	Stage	Site	Age dph	Weight mg
FLG	5	Flexion	Gr	24	5.9
MMG	2	Mid-metamorphosis	Gr	52	12
MMF	10	Mid-metamorphosis	Fr	50	48.9
MMI/EMI	3	Early metamorphosis	It	43	9.6
MMI/LMI	4	Late metamorphosis	It	58	33.1
EGG	1	-	Gr	-	10

The table is linked to associated research paper ([6], doi.org/10.1016/j.aquaculture.2024.740979); days post hatch (dph).

**Table 2:** Sequence statistics of 25 paired-end RNA-seq libraries mapped to the gilthead seabream reference genome.

Project	RNA-seq Library	Sample code*	Total Sequences (R1+R2)	Sequence length (R1/R2)	Total fragments	Total number of mapped	Proportion of mapped (%)
A	FLG1	FLG1	10.975.536	151	5487768	5291514	96.4
A	FLG2	FLG2	7.340694	151	3670347	3506422	95.5
A	FLG3	FLG3	6.100.328	151	3050164	2902469	95.1
A	FLG4	FLG4	21.564.414	151	10782207	10462493	97
A	FLG5	FLG5	19.660.540	151	9830270	9398255	95.6
A	MMG2	MMG2	9702012	151	4851006	4725268	97.4
B	MMI1	EMI1	43.697.894	151	21848947	21848947	97.1
B	MMI2	EMI2	42.225.904	151	21112952	20413870	96,7
B	MMI3	EMI3	54.395.422	151	27197711	25618116	94,2
A	MMG1	MMG1	8.616.928	151	4308464	4146651	96.2
A	MMF10	MMF10	21.408.178	151	10704089	10299343	96.2
A	MMF1	MMF1	10.111.324	151	5055662	4457975	88.1
A	MMF2	MMF2	8.455.506	151	4227753	4062694	96
A	MMF3	MMF3	9.638.312	151	4819156	4665511	96.8
B	MMF4	MMF4	44.503.122	151	22251561	21451485	96.4
B	MMF5	MMF5	45.500.268	151	22750134	22009768	96.7
B	MMI6	LMI6	45.452.800	151	22726400	20741904	91.3
B	MMI7	LMI7	44.873.170	151	22436585	20085755	89.5
B	MMI4	LMI4	43.197.300	151	21598650	20427004	94.6
B	MMI5	LMI5	56.233.626	151	28116813	26552801	94.4
B	MMF6	MMF6	47.886.108	151	23943054	22902195	95.7
B	MMF7	MMF7	43.037.428	151	21518714	20550522	95.5
B	MMF8	MMF8	47.873.228	151	23936614	22553328	94.3
B	MMF9	MMF9	44.173.844	151	22086922	20829119	94.3
B	EGG	-	46,188,936	151	23094468	22277235	96.5

⁎ Sample code links to the larval stages determined in the associated research paper [6].

**Table 3:** List of the top 40 up-regulated and down-regulated gene transcripts in gilthead seabream larvae at flexion (24 dph) compared to mid-metamorphosis (51 dph).

mRNA accession	logFC*	adj.P.Val	Gene ID	Gene name
XM_030401988.1	7.3	0.001178	115572156	apolipoprotein D-like
XM_030418545.1	5.7	0.015281	115582545	1,25-dihydroxyvitamin D(3) 24-hydroxylase, mitochondrial
XM_030429400.1	5.6	0.000319	115588642	snaclec A7-like
XM_030408177.1	5.6	0.000613	115575825	hemoglobin embryonic subunit alpha-like
XM_030416405.1	5.6	0.000134	115581370	protein SON-like
XM_030406226.1	5.6	0.000119	115574593	uncharacterized LOC115574593
XM_030401305.1	5.5	0.003152	115571743	transmembrane protease serine 9-like
XM_030430049.1	5.2	0.005615	115589236	nucleolin-like
XM_030394500.1	5.0	0.000999	115567679	F-box protein 32
XM_030401797.1	5.0	5.09E-05	115572036	actinodin2
XM_030421138.1	4.9	0.000206	115583901	green-sensitive opsin
XM_030406713.1	4.9	0.001312	115574988	hemoglobin embryonic subunit alpha-like
XM_030406719.1	4.8	0.00088	115574990	hemoglobin embryonic subunit alpha-like
XM_030393118.1	4.8	0.006206	115567002	hepcidin-like
XM_030407941.1	4.7	7.66E-06	115575704	myosin heavy chain, fast skeletal muscle-like
XM_030418973.1	4.7	0.005472	115582772	sodium- and chloride-dependent transporter B(0+)-like
XM_030410474.1	4.5	0.000298	115577598	uncharacterized LOC115577598
XM_030410751.1	4.5	0.040305	115577865	glutamate–tRNA ligase-like
XM_030422030.1	4.4	0.029602	115584540	complement C1q-like protein 2
XM_030434425.1	4.4	0.005253	115591979	myosin light chain kinase family, member 4b
XM_030418053.1	-6.9	0.02658	115582232	betaine–homocysteine S-methyltransferase 1-like
XM_030409421.1	-7.0	0.037554	115576841	secreted phosphoprotein 2
XM_030412818.1	-7.1	0.000928	115579304	C-type mannose receptor 2-like
XM_030425714.1	-7.2	0.04344	115586568	mucin-2-like
XM_030424815.1	-7.3	0.01899	115586055	cytochrome P450 2D15-like
XM_030407240.1	-7.4	0.001547	115575307	nuclear GTPase SLIP-GC-like
XM_030418228.1	-7.5	0.000669	115582314	heat shock protein beta-1-like
XM_030420793.1	-7.8	0.004294	115583685	retinoid-binding protein 7-like
XM_030432479.1	-7.9	0.002907	115590966	PLAC8-like protein 1
XM_030437833.1	-8.1	0.000134	115594063	ependymin
XM_030425271.1	-8.2	0.009304	115586325	gastricsin-like
XM_030408710.1	-8.2	0.025577	115576212	hemoglobin subunit beta-2-like
XM_030408717.1	-8.5	0.020845	115576218	hemoglobin subunit alpha-1
XM_030421712.1	-8.6	0.040912	115584354	fibroin heavy chain-like
XM_030414402.1	-8.7	0.000524	115580252	ATPase H+/K+ transporting subunit beta
XM_030422916.1	-8.7	0.016805	115584948	galactose-specific lectin nattectin-like
XM_030399702.1	-9.0	0.000255	115570906	pepsin A-like
XM_030422754.1	-9.1	0.042859	115584878	fibroin heavy chain-like
XM_030419000.1	-10.1	3.75E-05	115582787	acidic mammalian chitinase-like
XM_030422120.1	-10.9	1.20E-05	115584588	pepsin A-like

⁎ Positive values = up-regulated gene transcripts, negative (-) values = down-regulated gene transcripts, adj.P.Val = Benjamini and Hochberg (BH) method used to adjust the *p*-values.

**Table 4:** List of top 40 up-regulated and down-regulated gene transcripts in gilthead seabream larvae at mid-metamorphosis stage (46 dph) compared to late metamorphosis (54 dph).

mRAN accession	logFC*	adj.P.Val	Gene ID	Gene name
XM_030427745.1	5.1	0.02327	115587766	lactase-phlorizin hydrolase-like
XM_030406322.1	4.8	0.00824	115574657	apolipoprotein B-100-like
XM_030401430.1	4.7	0.01232	115571574	uncharacterized LOC115571574
XM_030420128.1	4.7	0.02123	115583368	Rh50-like protein
XM_030406431.1	4.6	0.02709	115574812	hemoglobin embryonic subunit alpha-like
XM_030425867.1	4.6	0.00449	115586658	peroxisomal succinyl-coenzyme A thioesterase-like
XM_030393725.1	4.6	0.01956	115567308	ectonucleotide pyrophosphatase
XM_030403895.1	4.5	0.00287	115573203	GTPase IMAP family member 9-like
XM_030413254.1	4.5	0.01346	115579674	uncharacterized LOC115579674
XM_030420597.1	4.5	0.01422	115583588	acidic mammalian chitinase-like
XM_030434995.1	4.4	0.01104	115592356	solute carrier family 34 member 2a
XM_030410396.1	4.4	0.00593	115577355	chloride intracellular channel 5a
XM_030394630.1	4.4	0.03009	115567784	uncharacterized LOC115567784
XM_030427746.1	4.4	0.01425	115587767	lactase-phlorizin hydrolase-like
XM_030399162.1	4.3	0.02039	115570587	mucin 13b, cell surface associated
XM_030437803.1	4.2	0.00705	115594043	beta-carotene oxygenase 2b
XM_030431943.1	4.2	0.00287	115590549	ubiquitin carboxyl-terminal hydrolase 17-like protein D
XM_030429506.1	4.2	0.01847	115588735	uncharacterized LOC115588735
XM_030410196.1	4.2	0.01197	115577221	solute carrier family 15 member 1b
XM_030394984.1	4.1	0.00655	115567997	antifreeze protein type IV
XM_030416535.1	-4.2	0.02103	115581454	myoglobin
XM_030415961.1	-4.3	0.00593	115581104	beta-1..beta-1,6-N-acetylglucosaminyltransferase-like
XM_030422916.1	-4.3	0.00574	115584948	galactose-specific lectin nattectin-like
XM_030422992.1	-4.4	0.0224	115584993	galactose-specific lectin nattectin-like
XM_030406979.1	-4.4	0.02039	115575113	cytochrome c oxidase subunit 6A, mitochondrial-like
XM_030394680.1	-4.5	0.03267	115567833	1-phosphatidylinositol phosphodiesterase-like
XM_030403961.1	-4.6	0.01525	115573251	uncharacterized LOC115573251
XM_030423689.1	-4.6	0.00626	115585370	galactose-specific lectin nattectin-like
XM_030423685.1	-4.7	0.00894	115585365	galactose-specific lectin nattectin-like
XM_030405178.1	-4.8	0.01723	115573994	transcobalamin beta a
XM_030399456.1	-4.8	0.01104	115570751	hemoglobin beta embryonic-2
XM_030422991.1	-5.0	0.0087	115584992	galactose-specific lectin nattectin-like
XM_030411086.1	-5.2	0.04522	115578220	neoverrucotoxin subunit alpha-like
XM_030433546.1	-5.5	0.00887	115591486	proproteinase E-like
XM_030411604.1	-5.5	0.00546	115578567	coagulation factor XI-like
XM_030438458.1	-5.5	0.03861	115594397	uncharacterized LOC115594397
XM_030422917.1	-5.9	0.0042	115584949	lactose-binding lectin l-2-like
XM_030423688.1	-5.9	0.01294	115585368	galactose-specific lectin nattectin-like
XM_030413465.1	-7.2	0.00676	115579796	microfibril-associated glycoprotein 4-like
XM_030399324.1	-8.8	0.00566	115570670	hemoglobin, alpha embryonic 5

⁎ Positive values = up-regulated gene transcripts, negative (-) values = down-regulated gene transcripts, adj.*P.Val* = Benjamini and Hochberg (BH) method used to adjust the *p*-values.

Supplementary Table 1 provides the Gene Set Enrichment Analysis results at the biological process (BP), molecular function (MF), and cellular component (CC) levels based on the obtained DEGs in gilthead seabream larvae at the flexion stage (24 dph) compared to mid-metamorphosis (51 dph). Supplementary Table 2 provides the Gene Set Enrichment Analysis results at the biological process (BP), molecular function (MF), and cellular component (CC) levels based on the obtained DEGs in gilthead seabream larvae at 46 dph (mid-metamorphosis stage) compared to 54 dph (mid-metamorphosis and bigger larvae). Supplementary Table 3 indicates the enriched pathways detected in KEGG Gene Set Enrichment analysis based on DEGs obtained when gilthead seabream larvae at the flexion stage (24 dph) were compared to mid-metamorphosis larvae (51dph). Supplementary Table 4 indicates the enriched pathways detected in KEGG Gene Set Enrichment analysis using the DEGs from gilthead seabream larvae at the mid-metamorphosis stage (46 dph) compared to the older larvae (54 dph).

## 4 Experimental Design, Materials and Methods

***Sample collection and RNA extraction*:** gilthead seabream larvae were collected from three hatcheries in France (Fr), Italy (It), and Greece (Gr) in 2018 (Fig. 1). The larvae collected from flexion, end of larval rearing, and metamorphosis stages, were fixed in RNA later, and kept at – 20°C until RNA extraction. Based on sample availability, two RNA-seq projects (A and B) were run to test transcriptome changes associated with the factors age and weight during the gilthead seabream larval development. Total RNAs (tRNA) from whole larvae were extracted using an E.Z.N.A. Total RNA Kit I (VWR, USA) according to the manufacturer's instructions. For the extractions two-three small larvae or one big larva was used for the RNA extractions (Table. 1). Larvae defrosted in the lysis buffer containing 20 µl β-mercaptoethanol per 1 ml of the TRK Lysis Buffer from the kit. The lysis buffer added was 350 µl for larval samples with a weight < 15 mg and 700 µl for larval samples with a weight > 15 mg. Larvae were homogenized by mechanical disruption with two iron beads (5 mm) using a Tissue lyser II Qiagen and three cycles (30 Hz) of 30 seconds at room temperature. The RNA quality was assessed using a 2100 Bioanalyser (Agilent) and quantified using an ND-2000 (NanoDrop Technologies, ThermoFisher). Only high-quality RNA samples (OD260/280 = 1.8 - 2.2, OD260/230 ≥ 2.0, RIN ≥ 8, 28S:18S ≥ 1.0, > 2 µg) were used to construct sequencing libraries.

***Library preparation, and Illumina Hiseq xten Sequencing:*** RNA-seq transcriptome libraries were prepared using a TruSeq TM RNA sample preparation Kit from Illumina (San Diego, CA) and 1 µg of total RNA (Novogene, Shanghai, China). Briefly, messenger RNAs were isolated using the poly-A selection method with oligo(dT) beads and were then fragmented using the supplied fragmentation buffer. Double-stranded cDNA was synthesized using a SuperScript double-stranded cDNA synthesis kit (Invitrogen, CA) with random hexamer primers (Illumina). The synthesized cDNA was subjected to end-repair, phosphorylation, and ‘A’ base addition as outlined in the Illumina's library construction protocol. The 200–300 bp cDNA fragments were isolated on 2% Low Range Ultra Agarose and were amplified in a PCR reaction (15 cycles) using Phusion DNA polymerase (NEB). After quantification using TBS380, paired-end RNA-seq libraries were sequenced using the Illumina HiSeq xten (2 × 150bp read length).

***Bioinformatics analysis:*** GALAXY FASTQC software was used to control the quality of the reads [1,2]. After quality control, the gilthead seabream reference genome index (Assembly name: fSpa Aur1.1, NCBI RefSeq assembly accession: GCF_900880675.1) was built using the Bioconductor package Rsubread and *buildindex* function in an R environment [4]. The reads were mapped to the reference genome using the align function and applying the default parameters. The total read number and proportion (%) of mapped reads were specified (Table 2). The number of paired-end reads mapped per gene was counted using the *featureCounts* function. Genes with a very low expression were filtered using the edgeR package and *cpm* function [7]. The read counts were normalized using the limma package and *voom* function. A multidimensional scaling plot/PCA of distances between gene expression profiles of larvae samples was performed using *plotMDS*. A single egg sample was also used in the analysis. The samples with a similar range of reads were compared together and used for downstream analysis (samples with an approximate total read number of 6-10 million for Project A, and samples with approximately 44-54 million of total reads from Project B). Samples with read numbers outside of the selected ranges (e.g., FLG4 with 21.5 million sequenced reads) and the outlier samples (e.g., MMG2 and EGG) were excluded from the differential gene expression (DEG) analysis to eliminate bias. DEGs were specified using the normalized data and comparing the larvae with an average age of 24 dph (flexion stage) versus 51 dph (mid-metamorphosis stage) and the larvae with an average age of 46 dph versus 54 dph (mid-metamorphosis stages, Table 3, Table 4, and for a fuller consideration of the results see, [6], https://doi.org/10.1016/j.aquaculture.2024.740979).

For functional analysis, orthologues of DEGs in each comparison were extracted from the zebrafish genome (assembly: GRCz11, GCA_000002035.4). Ensembl protein translation data of zebrafish was preformatted using the *makeblastdb* function in ubuntu v 20.04.2 LTS. DEG orthologues were extracted by blasting (ncbi-blast+) against the formatted zebrafish protein database using blastp in the Ubuntu environment and applying a cut-off for the *p-value* < 1E^−5^ and a sequence identity > 40 %. After removing the duplicated gene IDs, the resulting data was used in gene ontology and KEGG pathway analysis. The functional profile of the identified genes and pathways are interpreted in the associated research paper ([6], https://doi.org/10.1016/j.aquaculture.2024.740979) by selecting the GO terms or KEGG pathways supported by the Benjamini-Hochberg (BH) adjusted *p-value* method.

***Histological analysis:*** Based on the significant DE genes and processes identified from the transcriptome analysis of flexion and mid-metamorphosis stages of gilthead seabream, histology was carried out to characterize the development of organs and lipid deposition. Serial sagittal and transverse sections (10 µm thick) of paraffin wax embedded larvae (flexion= 23-25 dph, mid-metamorphosis = 50-56 dph) were prepared using a manual rotary microtome (Leica RM 2135, Germany). The morphological characteristics of organs during early development were characterized by staining sections with haematoxylin and eosin (H&E) using the method described in Najafpour et al. [5]. For detection of lipids, frozen sections were used and stained with Oil Red O using an adaptation of the methods reported in [3,8]. In brief, methanol fixed larvae were rinsed in phosphate-buffered saline (PBS, pH = 7.4) for two days, and then cryoprotected through a gradient (10%, 20 %, and 30%) of sucrose solutions in PBS (pH = 7.4, sodium chloride, 0.137 M; potassium chloride, 0.0027 M; sodium phosphate dibasic, 0.01 M; potassium phosphate monobasic, 0.0018 M), embedded in gelatine and snap frozen in dry ice. Serial sections of whole larvae were cut (10 µm thick) using a cryostat (Thermo Scientific) and mounted on 3-aminopropyltriethoxysilane (APES)-coated glass slides and were stored at -20°C until staining. For Oil Red O staining, the sections were dried at room temperature and immersed sequentially in 100% and 85 % isopropyl alcohol for 5 min. Then, the slides were immersed in a 0.5 % Oil Red O (Sigma-Aldrich, Madrid, Spain) solution for 4 h and then rinsed in distilled water. The slides were counterstained using haematoxylin (2 min) to visualize the nuclei and mounted in glycerine jelly aqueous mounting media. The slides were analysed using a microscope (Leica DM2000) and photographs were taken using a digital camera (Leica DFC480) linked to a computer.

## Limitations

Not applicable.

## Ethics Statement

Gilthead seabream larval samples were collected from hatcheries as part of their routine sampling regime to verify production performance and was carried out in compliance with 2009/58/EC (protection of animals kept for farming).

## CRediT Author Statement

**Babak Najafpour**: Investigation, Formal analysis, Methodology, Visualization, Writing-Original draft preparation, Reviewing. **Adelino VM Canário**: Writing, Reviewing and Editing. **Deborah M Power**: Supervision, Funding acquisition Writing-Original draft preparation, Reviewing and Editing.

## Data Availability

Transcriptomic datasets of the critical larval stages of Sparus aurata (Original data) (NCBI).

## Appendix Supplementary materials

Image, application 1

Image, application 2

Image, application 3

Image, application 4
